# Alcohol ingestion disrupts alveolar epithelial barrier function by activation of macrophage-derived transforming growth factor beta1

**DOI:** 10.1186/1465-9921-14-39

**Published:** 2013-04-02

**Authors:** Tiana V Curry-McCoy, Aida Venado, David M Guidot, Pratibha C Joshi

**Affiliations:** 1 Division of Pulmonary, Allergy, and Critical Care Medicine, Emory University School of Medicine, 615 Michael Street, Suite 205, Atlanta, GA, 30322-1047, USA; 2 Atlanta Veterans Affairs Medical Center, Decatur, GA, USA; 3 Current address: Georgia Regents University, Augusta, GA, USA; 4 Current address: University of Alabama, Birmingham, AL, USA

**Keywords:** Lung, Epithelial, Immune, Macrophage, Alcohol, Cytokines, Growth factors

## Abstract

**Background:**

Chronic alcohol abuse causes oxidative stress and impairs alveolar epithelial barrier integrity, thereby rendering the lung susceptible to acute edematous injury. Experimentally, alcohol-induced oxidative stress increases the expression of transforming growth factor β1 (TGFβ1) in the lung; however, we do not know the precise contribution of various alveolar cells in this process. In the present study, we focused on cell-cell interactions between alveolar macrophages and epithelial cells and the potential mechanisms by which TGFβ1 may become activated in the alveolar space of the alcoholic lung.

**Methods:**

Primary alveolar macrophages and epithelial cells were isolated from control- and alcohol-fed Sprague–Dawley rats. Expression of TGFβ1 and the epithelial integrin αvβ6 were examined by real time PCR and either immunocytochemistry or flow cytometry. Alveolar epithelial cells were cultured on transwell supports in the presence of macrophage cell lysate from control- or alcohol-fed rats or in the presence of viable macrophages ± alcohol. Epithelial barrier function was assessed by transepithelial resistance (TER) and paracellular flux of Texas Red dextran.

**Results:**

TGFβ1 expression was increased in alveolar macrophages from alcohol-fed rats, and TGFβ1 protein was predominantly membrane-bound. Importantly, alveolar macrophage cellular lysate from alcohol-fed rats decreased TER and increased paracellular dextran flux in primary alveolar epithelial cell monolayers as compared to the lysates from control-fed rats. Alcohol-induced epithelial barrier dysfunction was prevented by anti-TGFβ1 antibody treatment, indicating the presence of bioactive TGFβ1 in the macrophage lysate. In addition, co-culturing macrophages and epithelial cells in the presence of alcohol decreased epithelial barrier function, which also was prevented by anti-TGFβ1 and anti-αvβ6 treatment. In parallel, chronic alcohol ingestion *in vivo*, or direct treatment with active TGFβ1 *in vitro*, increased the expression of αvβ6 integrin, which is known to activate TGFβ1, in alveolar epithelial cells.

**Conclusions:**

Taken together, these data suggest that interactions between alveolar epithelial cells and macrophages contribute to the alcohol-mediated disruption of epithelial barrier function via the expression and activation of TGFβ1 at points of cell-cell contact.

## Background

Alcohol abuse impairs pulmonary innate immunity and renders individuals susceptible to pneumonia and lung injury [1]. One of the important features of lung injury is disruption of alveolar epithelial barrier function [2]. We have previously shown that chronic alcohol ingestion in rats increases oxidative stress [3] and transforming growth factor 1 (TGFβ1) expression in the lung [4], and inflammatory insults such as sepsis release activated TGFβ1 into the alveolar space, which can intensify lung injury by further disrupting alveolar barrier function. Ethanol ingestion decreases antioxidant glutathione in the alveolar space [5,6], and TGFβ1 is a potent inhibitor of glutathione synthesis in lung epithelial cells [7]. We have previously shown that ethanol ingestion also decreases another antioxidant, micronutrient zinc, in the lower airway [8] and adversely affects zinc importers through down-regulation of Kruppel-like transcription factor 4 by active TGFβ1 [9,10]. Thus, TGFβ1 alters these important antioxidants in the lung and impairs epithelial barrier integrity. In addition, TGFβ1 is immunosuppressant [11,12], and decreases immune function of alveolar macrophages by dampening GM-CSF receptors on these cells [13].

TGFβ1 protein has diverse and often contradicting biological activities regulating cell proliferation, differentiation, and function. TGFβ1 is synthesized as a latent complex and is present at the cell surface as latency-associated peptide (LAP) [14,15]. LAP is non-covalently linked to TGFβ and prevents binding of active TGFβ1 to its receptors. The cell-associated TGFβ1 is activated by a variety of stimuli like plasmin or thrombospondin. In addition, TGFβ1 can be activated by integrin receptors on epithelial cells. Integrins are heterodimers composed of α and β subunits and mediate cell interactions with other cells and with the extracellular matrix. The integrin αvβ6, initially identified as fibronectin receptor, is predominantly expressed by the epithelium lining of airways and alveoli [16] and is up regulated in response to injury. It is one of the LAP binding receptors, which binds to LAP resulting in conformational changes in LAP and exposes TGFβ1 residing within LAP to the TGFβ1 receptors on the adjacent cells. This initial binding of TGFβ1 to its receptor is a key step in TGFβ1 signaling and its multiple effects on various cells.

Cells often use signaling molecules such as hormones, cytokines, growth factors, and chemotactic factors to transmit signals and communicate with other cells. In endocrine system hormones, secreted by cells at one site, travel and bind to a receptor on a cell at a distant site. Within any organ, including the lung, signaling molecules such as cytokines/growth factors are released in the local environment and bind to signal-transducing receptor on the adjacent cell (paracrine signaling) or bind to receptors on itself (autocrine signaling), thus activating cells. In juxtacrine signaling, cytokine/growth factor is membrane-bound and activates adjacent cells after binding to receptors [12]. Thus, cell-cell communication in the alveolar compartment takes place in a paracrine, autocrine, or juxtacrine manner rather than in an endocrine manner. These types of cellular communications are important when examining the in vivo effects of a toxicant such as alcohol on the lung function.

The present study focuses on cell-cell interactions between alveolar macrophages and epithelial cells and extends our previous findings regarding TGFβ1’s role in disrupting epithelial barrier function. We evaluated the contribution of alveolar macrophages in alcohol-mediated impairment of alveolar epithelial barrier function. We report here that chronic alcohol ingestion increased TGFβ1 expression on alveolar macrophages. In parallel, alcohol ingestion increased integrin αvβ6 expression on epithelial cells. Importantly, co-culture of these cells in the presence of alcohol or culturing epithelial cells with macrophage lysates from alcohol-fed animals disrupted epithelial barrier function in a TGFβ1-dependent manner.

## Materials and methods

### Animals and alcohol feeding

Adult Male Sprague–Dawley rats (initial weights 150–200 g; Charles River Laboratory, Wilmington, MA) were fed the Lieber-DeCarli liquid diet (Research Diets, New Brunswick, NJ) containing either alcohol (ethanol; 36% of total calories) or an isocaloric substitution with maltose-dextrin *ad lib* for 6 weeks as previously published [17,18]. All work was performed with the approval of the Institutional Use and Care of Animals Committee at the Emory University.

### Brochoalveolar lavage and isolation of alveolar macrophages

Rats were anesthetized with 0.8 ml Euthasol containing penotobarbital sodium and phenytoin sodium (Vibac AH Inc, Fort Worth, TX). After pulmonary arterial perfusion, bronchial lavage was performed using 10mls of PBS 4 times and fluid was centrifuged at 405 g for 7 min to obtain alveolar macrophages. Cells were re-suspended in sterile F12-K complete medium containing antibiotics and 10% FBS for functional studies. This procedure routinely yields cells that are >98% viable by Trypan blue exclusion test [19].

### Isolation of primary alveolar type II epithelial cells

Alveolar epithelial cells from control- and alcohol-fed rats were isolated using our established protocol [6]. Briefly, lungs and trachea were removed as one unit and flushed with 40 ml of solution containing 16 mg of elastase. Lung lobes were cut and minced in a solution containing DNase I and newborn calf serum. The lung tissue suspension was shaken at 37°C for 10 minutes and filtered through 100 μm and 20 μm nylon mesh. The filtered lung suspension was then centrifuged at 405 g for 7 minutes, resuspended in 30 ml of complete medium containing DMEM/F12, antibiotics and fungicide, and plated on IgG coated dishes. Cells were incubated at 37°C 5% CO2 for 1 hour, and non-adherent cells were gently removed. Non-adherent type II cells were resuspended in complete medium and counted using a hemocytometer. Cell viability as determined by Trypan blue exclusion test was always >96%.

### Alveolar epithelial barrier function

Epithelial barrier function was examined by measuring transepithelial electrical resistance (TER) and determining paracellular permeability to Texas Red dextran (Invitrogen). Rat alveolar type II epithelial cells were plated at 50,000 per well in a 24 wells transwell plate. Cells were cultured in DMEM/F12 complete medium and treated the next day with alcohol (60 mM), anti-TGFβ1 Ab (1 μg/ml), anti-αvβ6 Abs (1 μg/ml), or IgG. Alcohol, antibodies or IgG were added with the replacement of fresh medium every other day to all the transwells. Transepithelial resistance was measured after 6 days using an epithelial voltohmmeter (World Precision Instruments, Sarasota, FL) as described before [20]. For paracellular permeability, sample transwells were placed in a plate containing 1 ml of 0.25 M-MgCl, 0.1 M-CaCl, PBS solution. Texas Red dextran (0.1 mg/ml) solution was added to the apical side of the monolayers in the transwell and basolateral samples were taken after 2 h incubation. The intensity of the dye was measured in a plate reader.

### Cell lines

In some experiments, rat lung epithelial cell line L2 (ATCC CCL-149, Manassas, VA) and rat alveolar macrophage cell line NR8383 (ATCC CCL-2192) were used. Cells were cultured in F12K with 10% FBS and an antibiotic-antimycotic reagent (Sigma-Aldrich, St Louis, MO) at 37°C in 5% CO_2_[21]. No TGFβ1 was detected in this culture medium containing FBS as measured by ELISA.

### RNA isolation and Real-time PCR

RNA was extracted from cells using Qiagen RNeasy Mini Kit (Valencia, CA). Reverse transcription was performed using 1 μg RNA using iScript cDNA synthesis kit from Bio Rad (Hercules, CA), and real time polymerase chain reaction was performed using primers for rat TGFβ1 (5^′^-TGAGTGGCTG-TCTTTTGACG-3^′^ and 5^′^-TGGGACTGATCCCATTGATT-3^′^), rat integrin chains αv (5^′^-GGGCATTTCAGGACTTGTGT-3^′^ and 5^′^-AGGTGACGGGACTCAAC-AAC-3^′^) & β6 (5^′^-AGGCCTGCTCTGTGGAGATA-3^′^ and 5^′^-CCATCTGC-AGACAGGTAGCA-3^′^) that were designed in our laboratory and obtained from Invitrogen (Carlsbad, CA). 18S Quantum RNA classic II primers were purchased from Ambion (Austin, TX). All samples were run in triplicate. Messenger RNA expression for each gene of interest was normalized to 18S housekeeping gene and then expressed as the change relative to the control group.

### Flow cytometric analysis

Flow cytometric analysis of membrane protein expression was performed using an established protocol in our laboratory [21]. Briefly, cells were not permeabilized and incubated with a primary polyclonal antibody or IgG (Santa Cruz biotechnology, Santa Cruz, CA) for one hour. Cells were washed with PBS, and stained with a PE-conjugated secondary antibody. The labelled cells were washed again with PBS and analyzed by FACScan flow cytometer (BD Bioscience, San Diego, CA). Data are expressed as percentage of cells positive for the protein.

### Immunofluorescence imaging

To a portion of the stained macrophages from the above protocol, Hoechst nuclear stain (Molecular Probes, Eugene, OR) was added. Cells were washed and put on slides to obtain images using a microscope equipped with epifluorescence (Olympus Corporation, Center Valley, PA).

### ELISA

TGFβ1 protein in the bronchoalveolar lavage and cells was measured using ELISA kit (BD biosciences).

### Statistics

Data are presented as mean ± SEM. Data analysis was performed by unpaired t test for two treatment groups and ANOVA with Student-Newman-Keuls test for group comparison for three or more treatment groups and was considered statistically significant at a value of *p* < 0.05.

## Results

### Chronic alcohol ingestion increased expression of TGFβ1 in alveolar macrophages

**Figure 1:**
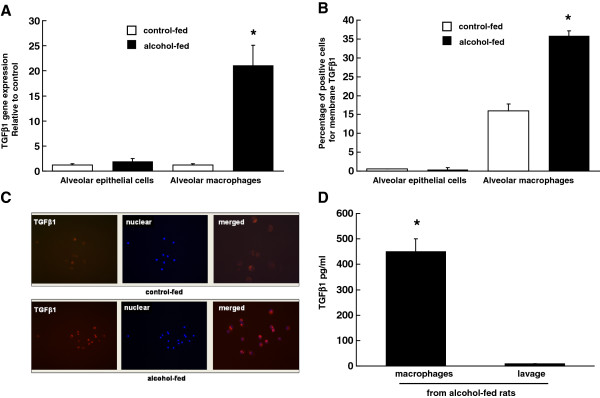
**Chronic alcohol ingestion increased expression of TGFβ1 in the alveolar macrophage.** (**A**) Gene expression of TGFβ1 in rat alveolar macrophages and epithelial cells. Cells were isolated from control- and alcohol-fed rats as described in the Methods. Gene expression of TGFβ1 was normalized to 18S. N = 3; * indicates p < 0.05 compared with macrophages from control-fed group. Each value represents the mean ± SEM. (**B**) Percentage of positive cells for membrane TGFβ1 as analyzed by flow cytometry in freshly isolated alveolar macrophages and epithelial cells from rats fed control- and alcohol-diet. (**C**) Immunofluorescence staining of alveolar macrophages from rats fed control- or alcohol-diet that were stained with anti-TGFβ1 antibody and Hoechst for nuclear staining. Control IgG did not show any staining. (**D**) TGFβ1 protein in the bronchoalveolar lavage fluid and cells from rats fed alcohol-diet was measured using ELISA as described in the Methods. N = 3; * indicates p < 0.05 compared with the lavage.

We used freshly isolated alveolar macrophages and epithelial cells from control- and alcohol-fed rats to examine the gene expression of TGFβ1. As shown in Figure 1; panel A, TGFβ1 gene expression in alveolar epithelial cells was low and there was no difference in the expression between cells from control- and alcohol-fed rats. In contrast, alveolar macrophages from alcohol-fed rats showed a significant increase in expression of TGFβ1 suggesting that chronic alcohol ingestion induces TGFβ1 in alveolar macrophages. Alveolar macrophages from alcohol-fed rats showed a significant increase in TGFβ1 gene expression as compared to cells from control-fed rats. Next, we examined TGFβ1 protein levels in macrophages by flow cytometry, immunochemistry, and ELISA. Alveolar macrophages were stained with either control IgG or anti-TGFβ1 followed by PE conjugated secondary antibody. The membrane expression (percentage of positive cells) of TGFβ1 was examined by flow cytometry. As shown in Figure 1; panel B, the percentage of positive cells for membrane TGFβ1 on macrophages from control- and alcohol-fed rats were significantly different (16.0 ± 1.8 and 35.8 ± 1.4, respectively; n = 5, p < 0.05). In contrast, epithelial cells had <1% cells positive for TGFβ1. Panel C in Figure 1 shows microscope images of macrophages positive for TGFβ1. These cells were stained without permeabalization and therefore show membrane-bound expression of TGFβ1 in cells from control- and alcohol-fed rats, and increased intensity of staining in cells from alcohol-fed rats. We have previously shown that bronchoalveolar lavage from alcohol-fed rats did not have secreted TGFβ1 [22]; however, we did not measure cellular TGFβ1. In the present study, Panel D shows Elisa measurement of TGFβ1 protein in the lysates of alveolar macrophages from alcohol-fed rats as compared to lavage. Cells showed a significantly higher production of TGFβ1 protein as compared to lavage suggesting that macrophages are the source of TGFβ1 in the alveolar space of alcohol-fed animals. Collectively the data from panels A-D suggest that the TGFβ1 gene expression was induced only in the alveolar macrophages by alcohol and significantly more TGFβ1 protein was present on macrophages from alcohol-fed rats as compared to controls.

### Bioactive TGFβ1 was present in the alveolar macrophage lysates from alcohol-fed rats

**Figure 2:**
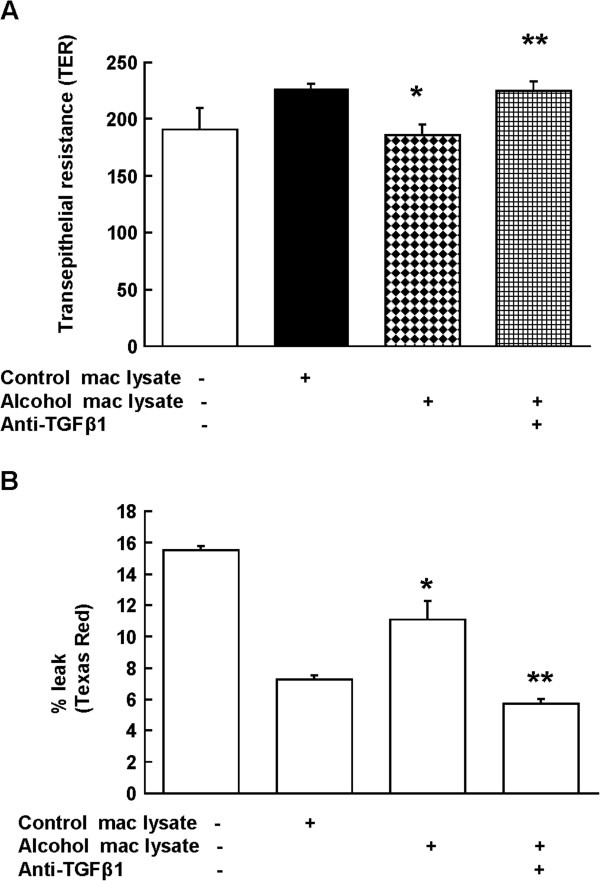
**Macrophage lysates from alcohol-fed rats impaired alveolar epithelial barrier function.** Freshly isolated alveolar epithelial cells from control-fed rats were cultured in transwells with or without cellular lysates of alveolar macrophages from alcohol-fed rats and anti-TGFβ1 Ab (1 μg/ml). The barrier function of epithelial monolayer was measured (**A**) by transepithelial resistance (TER) and (**B**) Texas Red dextran (10,000 MW) flux as described in the Methods. N = 5; * indicates p < 0.05 compared with lysates from control-fed rats in the co-culture group. ** indicates p < 0.05 compared with the group without anti-TGFβ1 Ab (third column).

Next, we examined whether or not TGFβ1 from alcoholic macrophages was bioactive. Alveolar macrophages (100,000) from control- and alcohol-fed rats were isolated and sonicated to obtain lysates. We added these cellular lysates to the transwells containing epithelial cells from control-fed rats, and examined changes in the epithelial barrier function after 72 h. The TER of the monolayer exposed to macrophage lysates from alcohol-fed rats was significantly lower than that of lysates from control-fed rats (Figure 2; panel A). Importantly, anti-TGFβ1 antibody significantly increased the TER to the control levels suggesting that alveolar macrophage lysates from alcohol-fed rats contained bioactive TGFβ1. Next, we added Texas Red dextran solution to the same plate and measured percentage leak of the dye in the bottom compartment. The transwells containing macrophage lysates from alcohol-fed rats showed significantly more leak as compared to those from control-fed rats or no lysates (panel B). This leak was prevented by anti-TGFβ1 antibody.

### Alcohol exposure decreased barrier function in alveolar epithelial cells co-cultured with alveolar macrophages via a TGFβ1-dependent mechanism

**Figure 3:**
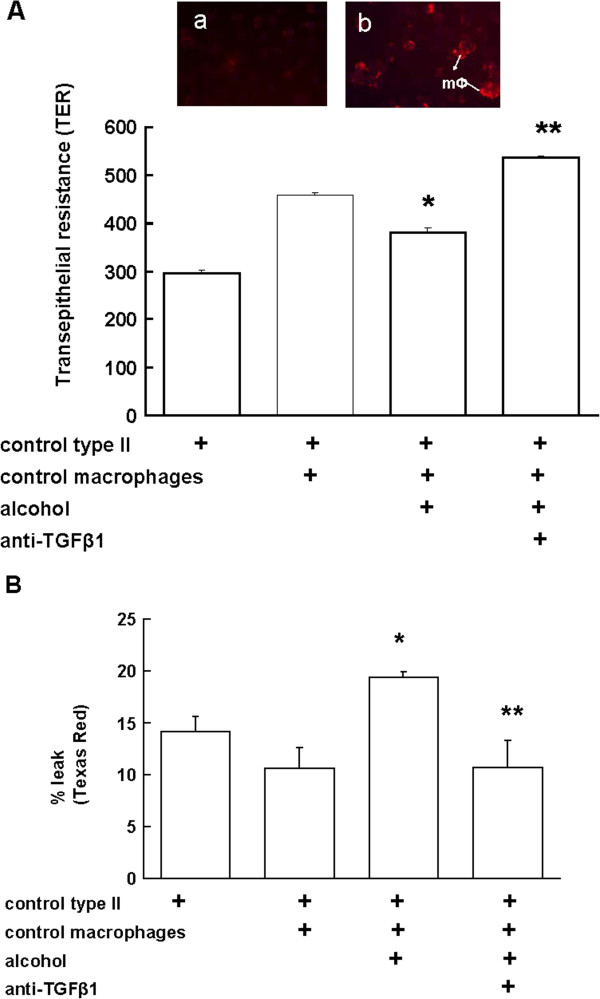
**Direct alcohol exposure decreased barrier function in alveolar epithelial cells co-cultured with macrophages.** Primary alveolar type II epithelial cells and macrophages from control-fed rats were cultured with or without alcohol or anti-TGFβ1 Ab (1 μg/ml) for 6 days. The barrier function of epithelial monolayer was measured (**A**) by transepithelial resistance (TER) and (**B**) Texas Red dextran (10,000 MW) flux as described in the Methods. TER was measured 3 times per sample and averaged per sample and per group (N = 5). * p < 0.05 compared to co-culture without alcohol (second column). ** p < 0.05 compared to co-culture with alcohol but without anti-TGFβ1 Ab (third column). Inset in (**A**) shows round macrophages stained positive for TGFβ1 in a co-culture of epithelial monolayer (no staining) and macrophages (stained on top) without alcohol (**a**) and in the presence of alcohol (**b**).

We have previously shown that type II epithelial cells from alcohol-fed rats have barrier dysfunction. Therefore, we examined if alcohol-induced TGFβ1 in macrophages contributed to epithelial barrier disruption. We co-cultured freshly isolated syngeneic alveolar macrophages and epithelial cells from control-fed rats and exposed these cells to 60 mM alcohol or medium for 6 days. Interestingly, epithelial cells co-cultured with macrophages formed tighter monolayers than epithelial cells cultured alone. The TER of monolayers after alcohol-exposure was significantly lower than monolayers without alcohol treatment (Figure 3; panel A), and it was restored after anti-TGFβ1 treatment. Next, we measured Texas Red flux in the same plate and found that alcohol treatment of co-cultured cells also increased the paracellular epithelial permeability as reflected by more dye on the basolateral side, and anti-TGFβ1 treatment also prevented this effect (Figure 3, panel B). No significant change in the flux was observed in the monolayers with or without macrophages. Inset in panel A shows round macrophages stained positive for TGFβ1 in a co-culture of epithelial monolayer (no staining) and macrophages (stained on top) in the presence of alcohol (b) as compared to without alcohol (a). These data suggest that cell-cell contact and the presence of alcohol are required for the disruption of epithelial barrier by TGFβ1 from co-cultured macrophages.

### The effect of alcohol on the expression of integrin chains αvβ6 on alveolar epithelial cells

**Figure 4:**
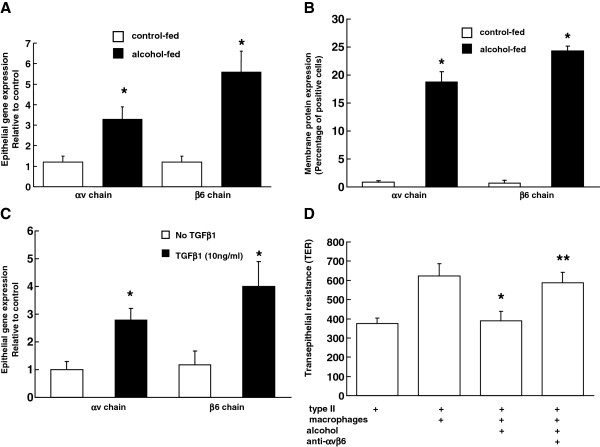
**Alcohol ingestion, or direct treatment with TGFβ1, increased the expression of the αvβ6 integrin in alveolar epithelial cells, and antibodies to αvβ6 reversed epithelial monolayer permeability in alcohol treated co-cultures of epithelial cells and macrophages.** Shown are (**A**) the relative gene expression, (**B**) flow cytometric analysis of membrane protein expression of the integrin chains αv and β6 in freshly isolated alveolar epithelial cells from control- and alcohol-fed rats, (**C**) the relative gene expression of αv and β6 in alveolar epithelial cells isolated from control-fed rats and treated *in vitro* with 10 ng/ml of active TGFβ1 for 48 h. n = 4; * p < 0.05 compared to control-fed in panel A & B and compared to untreated (no TGFβ1) group in panel C. In panel (**D**) primary alveolar type II epithelial cells and macrophages from four rats were cultured with or without 60 mM alcohol, anti-αvβ6 Abs (1 μg/ml) for 6 days. The barrier function of epithelial monolayer was measured by transepithelial resistance (TER) and data are shown as mean ± SEM. * p < 0.05 compared to co-culture without alcohol (second column). ** p < 0.05 compared to co-culture with alcohol but without antibodies to αvβ6 (third column). Transepithelial resistance for alcohol treated co-cultures with anti-TGFβ1 antibody and IgG in this experiment was 637 ± 29 and 465 ± 57, respectively (not shown in the graph).

To examine if alveolar epithelial cells from alcohol-fed rats are just the target of TGFβ1-mediated disruption of barrier function or contribute to their injury, we focused on the expression of integrin chains αv and β6 on epithelial cells. Integrins are a large family of heterodimeric transmembrane glycoproteins and are expressed by many cell types. Integrin αvβ6 was initially identified as a receptor for fibronectin, and its expression is restricted to epithelial cells in the airways and alveoli. As shown in the Figure 4; panels A & B, freshly isolated alveolar epithelial cells from alcohol-fed rats showed a significantly higher gene and protein expression of αv and β6 as compared to cells from control-fed animals. In preliminary experiments with co-cultures of epithelial (L2) and macrophage (NR8383) cell lines we found a simultaneous ~8 and ~32 fold increase in αv and TGFβ1, respectively after alcohol treatment (data not shown; n = 3), and the increase in αv in the co-cultures was higher than L2 cells in the presence of alcohol. This suggested a possibility that active TGFβ1 in the co-cultures may have some effect on the expression of αv. As shown in Figure 4; panel C, treatment of primary epithelial cells by active TGFβ1 for 48 h modestly but significantly increased the expression of integrin chains αv and β6. Together, these data suggests that alcohol ingestion in vivo or in vitro treatment with active TGFβ1 induce the expression of αvβ6 on alveolar epithelial cells suggesting a feedback regulation by alcohol-induced TGFβ1 on integrin expression. Next, we co-cultured epithelial cells and syngeneic macrophages from control rats (as in Figure 3A) and treated them in vitro with alcohol with or without αvβ6 antibodies or IgG for six days. Treatment with αvβ6 antibodies significantly increased TER of the monolayers as compared to those without antibodies suggesting that integrity of epithelial monolayers depends on alcohol-induced expression of αvβ6 and their activation of TGFβ1 on macrophages.

## Discussion

We previously reported that chronic alcohol ingestion in an experimental rat model increased TGFβ1 and that this is associated with alcohol-mediated epithelial dysfunction [22]. Further, although there was no evidence of TGFβ1 release into the alveolar space during baseline or ‘unstressed’ conditions, there was a marked increase in the activation and release of TGFβ1 into the alveolar space in response to acute endotoxemia, further enhancing alveolar epithelial barrier disruption [22]. These experimental findings suggested that this could be a contributing mechanism underlying the strong association between alcohol abuse and an increased risk of acute lung injury [23]. In the present study, although we again determined that TGFβ1 is not activated and released into the extracellular fluid spontaneously, it is activated *in situ* in the non-septic alcoholic lung. This mechanism could explain why even in the absence of an acute stress such as sepsis there is nevertheless alveolar epithelial barrier dysfunction even in the otherwise healthy experimental animals during chronic alcohol ingestion as reflected by increased paracellular leak of radiolabeled albumin [6]. We show here that (1) alveolar macrophages from alcohol-fed rats have increased expression of TGFβ1 and that the TGFβ1protein is membrane-bound, (2) co-culture of alcohol-primed macrophages and epithelial cells disrupted alveolar epithelial barrier function in a αvβ6- and TGFβ-dependent manner, (3) treating alveolar epithelial cells with the lysates of alveolar macrophages from alcohol-fed rats decreased their barrier function and this effect was antagonized by co-treating with an anti-TGFβ1 antibody, (4) alveolar epithelial cells from alcohol-fed rats had increased expression of the integrin chains αv and β6, and (5) treating alveolar epithelial cells with active TGFβ1 *in vitro* also increased the expression of these integrin chains, suggesting a forward feedback in which TGFβ1 induces the expression of the integrin that activates it.

The lung is comprised of multiple cell types including alveolar epithelial cells and macrophages in close vicinity, and cells communicate with each other, either in a paracrine manner through locally secreted cytokines/growth factors, or in a juxtacrine manner via cell-associated cytokines or growth factors. We identified membrane-bound TGFβ1 on alveolar macrophages suggesting a juxtacrine interaction with adjacent epithelial cells. Whether TGβ1 is stored in the extracellular matrix or on the surface of alveolar macrophages, it is present as a latent complex within a prodomain that shields it from binding to its receptors. The binding of the αv chain to an RGD sequence in the prodomain and exertion of force on this domain changes its conformation and activates TGFβ1, which can then bind to TGFβ receptors and initiate a wide range of intracellular signals.

Cell surface integrins regulate cell growth, migration, and survival. The αvβ6 integrin is a transmembrane glycoprotein that is mainly expressed by injured epithelium [24]. Integrins participate in activation of growth factors and initiate intracellular signaling cascades in response to receptor binding [24]. The integrin αvβ6 binds to the latency-associated peptide leading to activation of TGFβ1 [25]. Studies in αvβ6 knockout mice showed a deficiency in TGFβ1 activation by the epithelium and increased inflammation in response to injury and infection. Further, transgenic mice with a targeted deletion of the β6-integrin developed exaggerated lung inflammation [26] that was prevented by restoring β6 expression. Interestingly, bleomycin treatment leads to lung fibrosis due to increased activation of TGFβ1 [27]. *In vivo*, the αvβ6 integrin is an activator of TGFβ1, which stimulates fibroblast proliferation and collagen production and has been implicated in fibrosis [28].

TGFβ1 is known to regulate many biological processes. Cells produce TGFβ1 as a latent complex and the active peptide must be released from this complex in order to be activated and bind its receptors. Activation of TGFβ1 within the epithelium by the αvβ6 integrin plays a role in many diseases [29], including airway hyperresponsiveness in allergic asthma [30]. Other integrins such as αvβ5 are implicated in TGFβ1 activation in myofibroblast differentiation in fibrotic lungs [31]. In contrast to stress fibers used by fibroblasts and other contractile cells, epithelial cells exert force on latent TGFβ using actin/myosin [29]. Nevertheless, in both cell types mechanotransducers are involved in TGFβ1 activation. In idiopathic pulmonary fibrosis, the lung epithelium plays a key role in the fibrotic response and integrin-mediated activation of TGFβ1 has been implicated as a primary driver of this pathophysiology [32]. In fact, the activation of TGFβ1 by αvβ6 has been proposed as a potential therapeutic target for fibrotic lung diseases. In experimental model of airway fibrosis Mitchell et al. showed that chronic alcohol ingestion was associated with amplification of airway fibrosis through increase in IL-13 signalling [33]. Interestingly, IL-13 modulates TGFβ1 signalling during airway fibrosis, and alcohol’s priming effect for increased IL-13 signalling may play a role in lung transplantation related injury. Integrin αvβ6 plays a role in acute lung injury induced by *Pseudomonas aeruginosa*[34] and deletion of this integrin provides protection in experimental models of lung injury due to bleomycin or high tidal volume ventilation. Lung biopsies from patients with a diagnosis of IPF show staining for integrin αvβ6 within pneumocytes [35], and partial inhibition of TGFβ using integrin αvβ6 antibodies was effective in blocking murine pulmonary fibrosis without inducing an inflammatory response. Beta 6 integrin expression increased within the alveolar epithelium in radiation induced fibrosis model [36] and anti-αvβ6 therapy prevented fibrosis. Many αv integrins play a role in preventing inappropriate vascular growth and controlling vascular permeability, and studies in mice lacking the beta 6 subunit found a role for integrin-mediated TGFβ1 activation in pulmonary and renal fibrosis, acute lung injury, and pulmonary emphysema [37]. These studies elucidate the important potential contributions of αvβ6-mediated activation of TGFβ1 in many diseases.

Cytokines such as TGFβ1 have multiple and often diverse functions on different cell types. Activated TGFβ1 in situ can disrupt epithelial cell function and barrier integrity causing edema. In addition, active TGFβ1 can favor proliferation of fibroblasts and this has potential to lead to fibrosis or can decrease immune function of macrophage [13]. Thus, alcohol’s damaging effects on the lung involve multiple cell types and interactions between them. One of the mechanisms by which chronic alcohol abuse leads to oxidative stress includes activation of renin-angiotensin system in the lung. Alcohol-induced amplification of the renin-angiotensin system appears to be the major cause of the alveolar epithelial oxidant stress and TGFβ1-mediated barrier disruption [22]. Examining cell-cell interactions in the lung such as done in the current study may discover, in future, interconnected pathophysiological targets for alcohol abuse.

## Conclusion

**Figure 5:**
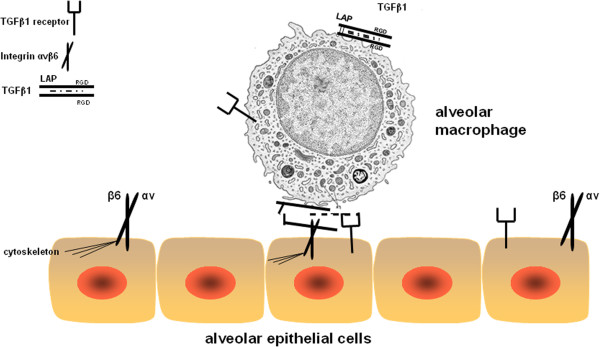
**Schematic representation of the proposed hypothesis.** Shown here is TGFβ1 pro-protein on the membrane. Inside (dashed line) is active TGFβ1 non-covalently bound to LAP and thus unable to bind to TGFβ1 receptors, which transduce signal. In the alcoholic lung, the expression of integrin αvβ6 is increased on epithelial cells, which binds to LAP on the alveolar macrophage. Because of the conformational change in the LAP, TGFβ1 slides out and is able to bind to the TGFβ1 receptors on the epithelial cells. This leads to decreased alveolar epithelial type II barrier function.

In summary, this study shows that chronic alcohol ingestion in rodents increases the expression of membrane-bound TGFβ1 on alveolar macrophages and simultaneously induces the expression of the αvβ6 integrin on alveolar epithelial cells. Alcohol treatment directly on these two cell types in co-culture disrupted alveolar epithelial barrier function in a TGFβ1-dependent manner. Although the exact mechanisms by which TGFβ1 disrupts epithelial barrier function in the alcoholic lung are not clear, it appears that direct interaction between alveolar macrophages and epithelial cells at their surfaces activates and releases TGFβ1 at low levels in the chronic state and at very high levels during sepsis or other inflammatory stresses. This aberrant expression and activation of TGFβ1 in both the chronic ‘unstressed’ state and the acutely ‘stressed’ state may explain many of the pathophysiological features that characterize the ‘alcoholic lung’ and its susceptibility to infection and injury (Figure 5).

## Competing interests

The authors declare that they have no competing interests.

## Authors’ contributions

TCM conducted experiments, collected and analyzed data, and drafted the manuscript. AV conducted experiments, collected and analyzed data. DMG provided intellectual content and editorial support. PCJ designed the study, conducted experiments, collected and analyzed data, and helped to draft the manuscript. All authors read and approved the final manuscript.
